# Ball Milling Assisted Solvent and Catalyst Free Synthesis of Benzimidazoles and Their Derivatives

**DOI:** 10.3390/molecules21091111

**Published:** 2016-08-24

**Authors:** Taghreed H. EL-Sayed, Asmaa Aboelnaga, Mohamed Hagar

**Affiliations:** 1 Faculty of Science, Chemistry Department, Yanbu, Taibah University, Yanbu, Saudi Arabia; taghreed.hussien@yahoo.com (T.H.E.); asmaa_aboelnaga77@yahoo.com (A.A.); 2 Faculty of Women for Arts, Science and Education, Chemistry Department, Ain Shams University, Heliopolis, Cairo 11757, Egypt; 3 Faculty of Science, Chemistry Department, Alexandria University, Alexandria 21321, Egypt

**Keywords:** Ball Milling, mechanochemistry, green chemistry, combinatorial chemistry, benzimidazoles

## Abstract

Benzoic acid and *o*-phenylenediamine efficiently reacted under the green solvent-free Ball Milling method. Several reaction parameters were investigated such as rotation frequency; milling balls weight and milling time. The optimum reaction condition was milling with 56.6 g weight of balls at 20 Hz frequency for one hour milling time. The study was extended for synthesis of a series of benzimidazol-2-one or benzimidazol-2-thione using different aldehydes; carboxylic acids; urea; thiourea or ammonium thiocyanate with *o*-phenylenediamine. Moreover; the alkylation of benzimidazolone or benzimidazolthione using ethyl chloroacetate was also studied.

## 1. Introduction

Ball Milling is a green method where reagents are placed inside a vessel with grinding balls that is shaken at high speeds in the absence of solvent [1,2,3]. The high speed is enough force to facilitate a chemical reaction which makes an amorphous mixture of the reagents. Recently, this method has been used in the generation of inorganic salts [4,5,6,7] while this methodology is relatively new to organic synthesis. The mechanochemical process is a separate branch of chemistry because chemical reactions that are caused by mechanical processes work directly and this kind of reaction is often different from conventional heating. 

Although ball milling organic synthesis is relatively new, the absence of solvents in the reaction is one of the advantages of ball milling technology, a tool with fast growing fields of application.

One of the most biologically active classes of compounds is benzimidazoles and its derivatives, which are well-documented in literature possessing a wide spectrum of activities. They show selective neuropeptides YY1 receptor antagonists [8,9,10,11], excellent inhibitors of TIE-2 and VEGFER-2 tyrosine kinase receptors [12,13,14,15,16,17,18], antitumor agents [19,20,21], gamma-amino butyric acid (GABA) agonists, and 5-HT3 antagonists [22,23,24,25].

Continuing of our interest [26,27,28] in finding a facile, efficient and green method for preparation of nitrogen heterocycles, herein, the aim of this work is to investigate an efficient green ball milling solvent-free synthesis of a series of benzimidazoles in good yield. The outcome of a chemical reaction in a ball mill mainly depends on the amount of energy that is supplied. Several reaction parameters directly influence this energy input. Some of these parameters are rotation frequency, milling balls weight and milling time.

## 2. Results and Discussion

### 2.1. Influence of the Reaction Parameters

The reaction parameters influence the energy input directly and can be changed and controlled during ball milling. These significant parameters include: variables frequency, milling balls weight and milling time. It could be proven that these three factors are the main ones impacting organic reactions in ball mills [29,30,31].

#### 2.1.1. Influence of Rotation Frequency

**Figure 1:**
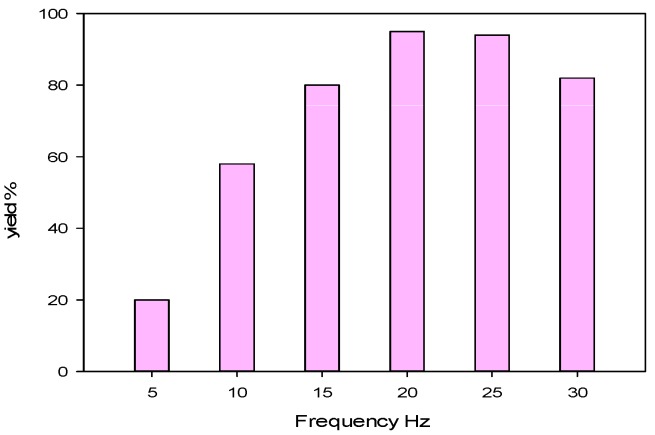
Milling of 2.5 g of *o*-phenylenediamine and 3 g of benzoic acid with 56.6 g of balls for 60 min.

The effect of the rotation frequency was investigated in a range from 5–30 Hz. The results (Table 1, Figure 1) are confirm that, as the rotation frequency rises, the yield of the reaction increases due to increased kinetic energy of the molecules and increased collision between the substrates. The increase in the yield of the reaction continues up to a point, “the optimum rotation frequency” (20 Hz), a maximum yield of product (95%) beyond which any further increase in a rotation frequency causes a decrease in the yield of the reaction. 

#### 2.1.2. Influence of Milling Balls Weight

**Figure 2:**
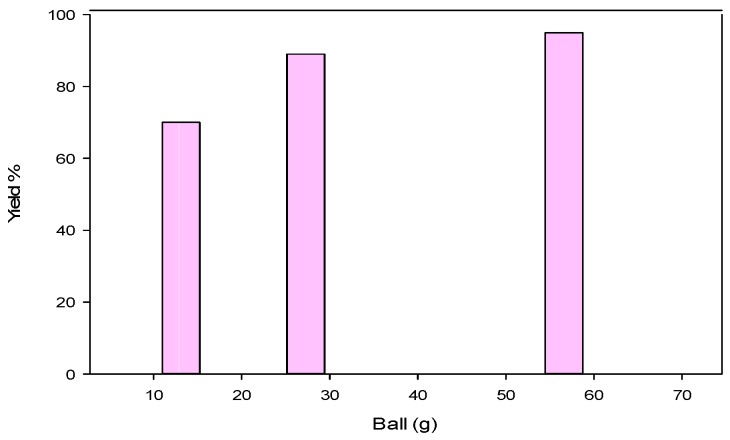
Milling of 2.5 g of *o*-phenylenediamine and 3 g of benzoic acid at 20 Hz for 60 min.

Technological parameters describe the engineering part of variables that can influence the outcome of a reaction, one of these parameters is the weight of the milling balls. The results show that the yield of the reaction product increases as the weight of milling balls increases (Table 2, Figure 2). A total of 14.4 g of milling balls weight affords only 70% of bezamidazole product after 60 min milling time, while 56.60 g increases the overall yield to 95%.

#### 2.1.3. Influence of Milling Time 

**Figure 3:**
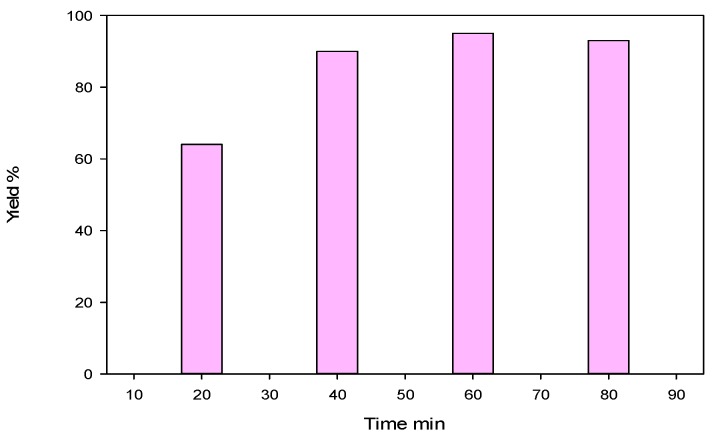
Milling of 2.5 g of *o*-phenylenediamine and 3 g of benzoic acid with 56.6 g of balls at 20 Hz.

To obtain high yields of the product in a short reaction time, milling conditions were optimized. The optimization of reaction time was necessary for completion of the reaction and to avoid by-products. The reaction progress was evaluated by observing the reaction mixture at various times (Table 3, Figure 3). The maximum yield of 95% was obtained by 60 min milling time at milling frequency 20 Hz and using 56.60 g milling balls. The prolonged reaction time is not preferable (80 min), and the yield decreased to 93% instead of 95% for a shorter time (60 min). 

Several reactions were carried out on *o*-phenylenediamine and different organic substrate as the model by varying the reaction time and finally the best result was obtained. The Milling of *o*-phenylenediamine and benzaldehyde with 56.6 g of balls at 20 Hz gave a maximum yield (97%) at 60 min. Longer milling time does not cause any increase in the yield (Table 4).

Also, the milling of *o*-phenylenediamine and of urea with 56.6 g of balls at 20 Hz causes the overall yield of reaction to increase with increasing time until reaching maximum value (73%) at 80 min (Table 5).

Twenty minutes of milling time of benzimidazol-2-one, sodium hydride and ethyl chloroacetate with 56.6 g of balls at 20 Hz affords the alkylating product at 96% yield. The increase in the milling time to 30 min did not affect in the amount of yield (Table 6)

**Scheme 1:**
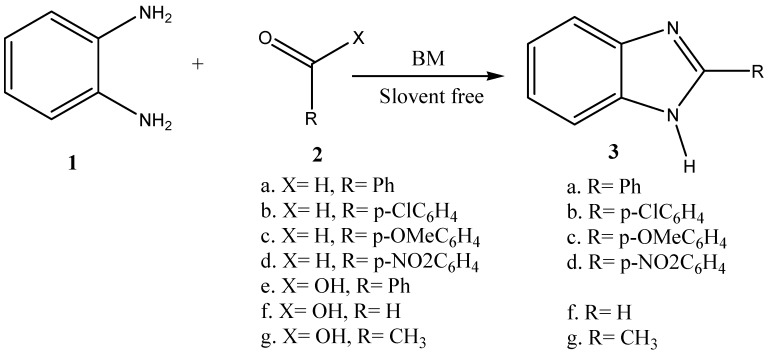
Synthesis of various functionalized 2-substituted benzimidazole.

**Scheme 2:**
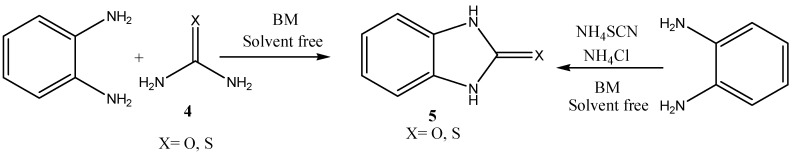
Synthesis of benzimidazol-2-one and benzimidazol-2-thione.

Optimum conditions was examined by subjecting different aldehydes, carboxylic acids, urea thiourea or ammonium thiocyanate with *o*-phenylenediamine with 56.6 g of balls for 60 min at 20 Hz. Various functionalized products (Scheme 1 and Scheme 2) were obtained in excellent selectivity and yields (Table 7).

**Scheme 3:**
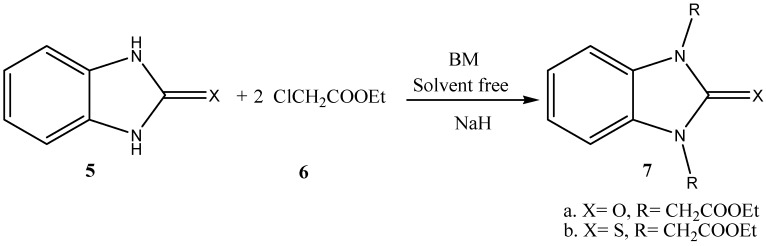
Alkylation of benzimidazol-2-one and benzimidazol-2-thione.

Milling of benzimidazol-2-one or benzimidazol-2-thione with ethyl chloroacetate in presence of sodium hydride with 56.6 g of balls for 20 min at 20 Hz, affords the alkylating product (**7a**,**b**) in high yield (Scheme 3, Table 8).

The ball milling method offers several advantages such as high conversions, shorter reaction times, non-toxic cost efficiency providing, cleaner reaction profiles and simple experimental and work-up procedures. In summary, a simple work-up procedure, mild reaction conditions and very good yields make our methodology a valid contribution to the existing processes in synthesis of benzimidazole derivatives.

## 3. Materials and Methods 

### 3.1. General Method

The ball mill was a SPEX 8000 mixer with 250 cm^3^ stainless steel vials. TLC were performed on Merck Kiesel gel; 60-F254 plates, and the spots were detected by UV light absorption.

### 3.2. General Reaction of o-Phenylenediamine with Carboxylic Acids or Aldehydes

A mixture of 1,2-phenylenediamine (2.5 g, 23.1 mmol) and 27.2 mmol of the appropriate carboxylic acid or aldehyde were introduced into stainless steel vials with appropriate weight of stainless steel balls for the mentioned time. The reaction was poured into water and neutralized with solid sodium carbonate. The formed precipitate was filtered, washed with water and recrystallized from ethanol-water.

### 3.3. General Reaction of o-Phenylenediamine with Ammonium Thiocyanate, Urea and Thiourea

A mixture of 1,2-phenylenediamine (2.5 g, 23.1 mmol) and 25.0 mmol of ammonium thiocyanate, urea or thiourea was introduced into stainless steel vials with appropriate weight of stainless steel balls for the mentioned time. The reaction was poured into water. The formed precipitate was filtered, washed with water and recrystalized from ethanol. 

### 3.4. General Alkylation Reaction of Benzimidazol-2-one and Benzimidazol-2-thione

A mixture of (2.5 g) of benzo[*d*]imidazol-2-one or benzimidazol-2-thione, 2 g of sodium hydride and 2.2 equivalent of ethyl chloroacetate was introduced into stainless steel vials with appropriate weight of stainless steel balls for the mentioned time. The reaction mixture was added to 100 g of ice-water. The precipitate was collected by filtration, washed with water and ethanol and recrystalized from hot ethanol.

## 4. Conclusions

In conclusion, a green solvent-free Ball Milling method for preparation of benzimidazole derivatives was studied under variable reaction parameters. The best reaction condition was 56.6 g weight of balls at 20 Hz frequency for one hour milling time to give ~95% of product yield. A high yield (64–97%) of benzimidazol-2-one derivatives was synthesized from different aldehydes, carboxylic acids, urea thiourea or ammonium thiocyanate with *o-*phenylenediamine. Moreover, dialkylation of benzimidazolone or benzimidazolthione using ethyl chloroacetate proceeded to give the corresponding product in 97% and 83%, respectively.

## Figures and Tables

**Table 1:** Milling of 2.5 g of *o*-phenylenediamine and 3 g of benzoic acid with 56.6 g of balls for 60 min.

Entry	Frequency (Hz)	% Yield
1	5	20
2	10	58
3	15	80
4	20	95
5	25	94
6	30	82

**Table 2:** Milling of 2.5 g of *o*-phenylenediamine and 3 g of benzoic acid at 20 Hz for 60 min.

Entry	Balls (g)	No of Balls (mm diameter)	% Yield
1	14.12	1 (12.7 mm), 1 (6.3 mm), 1 (3.2 mm)	70
2	28.27	2 (12.7 mm), 2 (6.3 mm), 2 (3.2 mm)	89
3	56.60	4 (12.7 mm), 4 (6.3 mm), 4 (3.2 mm)	95

**Table 3:** Milling of 2.5 g of *o*-phenylenediamine and 3 g of benzoic acid with 56.6 g of balls at 20 Hz.

Entry	Time (min)	% Yield
1	20	64
2	40	90
3	60	95
4	80	93

**Table 4:** Milling of 2.5 g of *o*-phenylenediamine and 2.7 g of benzaldehyde with 56.6 g of balls at 20 Hz.

Entry	Time (min)	% Yield
1	20	41
2	40	88
3	60	97
4	80	97

**Table 5:** Milling of 2.5 g of *o*-phenylenediamine and 1.5 g of urea with 56.6 g of balls at 20 Hz.

Entry	Time (min)	% Yield
1	20	20
2	40	55
3	60	72
4	80	73

**Table 6:** Milling of 2.5 g of benzimidazol-2-one, 2 g sodium hydride and 5.0 g of ethyl chloroacetate with 56.6 g of balls at 20 Hz.

Entry	Time (min)	% Yield
1	10	63
2	20	96
3	30	96

**Table 7:** Milling of 2.5 g of o-phenylenediamine and 3 g of aldehyde, carboxylic acids, urea or ammonium thiocyanate with 56.6 g of balls for 60 min at 20 Hz.

Product	Reactant	m.p. (°C)	% Yield
**3a**	Benzaldehyde	294 [32]	97
	Benzoic acid		95
**3b**	*p*-Chlorobenzaldehyde	302 [33]	91
**3c**	*p*-Methoxybenzaldehyde	225 [33]	80
**3d**	*p*-Nitrobenzaldehyde	312 [33]	96
**3e**	Benzoic acid	294 [33]	95
**3f**	Formic acid	170 [34]	89
**3g**	Acetic acid	177 [35]	80
**5a**	Urea	100 [36]	72
**5b**	Thiourea	307 [36]	67
	Ammonium thiocyanate		64

**Table 8:** Milling of 2.5 g of benzimidazol-2-one or benzimidazol-2-thione, 2 g of sodium hydride and 2.2 equivalent of alkylating agent with 56.6 g of balls for 20 min at 20 Hz.

Product	Substrate	Alkylating Agent	m.p. (°C)	% Yield
**7a**	Benzimidazol-2-one	Ethyl chloroacetate	177 [37]	97
**7b**	Benzimidazol-2-thione	Ethyl chloroacetate	200 [37]	83

## References

[1] James S., Adams C., Bolm C., Braga D., Collier P., Friscic T., Grepioni F., Harris K., Hyett G., Jones W. (2012). Mechanochemistry: Opportunities for new and cleaner synthesis. Chem. Soc. Rev..

[2] Stolle A., Szuppa T., Leonhardt S.E.S., Ondruschka B., Leonhardt S. (2011). Ball milling in organic synthesis: Solutions and challenges. Chem. Soc. Rev..

[3] Wang G.W. (2013). Mechanochemical organic synthesis. Chem. Soc. Rev..

[4] Zhang J., Riesen H. (2015). Mechanochemical preparation of nanocrystalline NaYF4: Gd^3+^/Yb^3+^/Tm^3+^: An efficient upconversion phosphor. Chem. Phys. Lett..

[5] Wang X., Riesen H. (2015). Mechanochemical synthesis of an efficient nanocrystalline BaFBr: Eu^2+^ X-ray storage phosphor. RSC Adv..

[6] Wang X., Liu Z., Stevens-Kalceff M.A., Riesen H. (2014). Mechanochemical preparation of nanocrystalline BaFCl doped with samarium in the 2+ oxidation state. Inorg. Chem..

[7] Zheng Z.G., Zhong X.C., Zhang Y.H., Yu H.Y., Zeng D.C. (2008). Synthesis, structure and magnetic properties of nanocrystalline ZnxMn1-xFe_2_O_4_ prepared by ball milling. J. Alloys Comp..

[8] Zhuang Z.P., Kung M.P., Chumpradit S., Mu M., Kung H.F. (1994). Derivatives of 4-(2′-methoxyphenyl)-1-[2′-(*N*-2″-pyridinyl-*p*-iodobenzamido) ethyl]piperazine (*p*-MPPI) as 5-HT1A ligands. J. Med. Chem..

[9] Kim J.S., Gatto B., Yu C., Liu A., Liu L.F., la Voie E.J. (1996). Structure-activity relationships of benzimidazoles and related heterocycles as topoisomerase I poisons. J. Med. Chem..

[10] Chimirri A., Grasso S., Monforte P., Rao A., Zappalà M., Monforte A.M., Pannecouque C., Witvrouw M., Balzarini J., de Clercq E. (1999). Synthesis and biological activity of novel 1*H*,3*H*-thiazolo[3,4-*a*]benzimidazoles: non-nucleoside human immunodeficiency virus type 1 reverse transcriptase inhibitors. Antivir. Chem. Chemother..

[11] Horton D.A., Bourne G.T., Smythe M.L. (2003). The combinatorial synthesis of bicyclic privileged structures or privileged substructures. Chem. Rev..

[12] Gravatt G.L., Baguley B.C., Wilson W.R., Denny W.A. (1994). DNA-directed alkylating agents. 6. Synthesis and antitumor activity of DNA minor groove-targeted aniline mustard analogues of pibenzimol. J. Med. Chem..

[13] Jayashankara B., Rai K.M.L. (2008). Synthesis and evaluation of antimicrobial activity of a new series of bis(isoxazoline) derivatives. Arkivoc.

[14] Roth T., Morningstar M.L., Boyer P.L., Hughes S.H., Buckheit R.W., Michejda C.J. (1997). Synthesis and biological activity of novel nonnucleoside inhibitors of HIV-1 reverse transcriptase. 2-Aryl-substituted benzimidazoles. J. Med. Chem..

[15] Lin S.N., Yang L.H. (2005). A simple and efficient procedure for the synthesis of benzimidazoles using air as the oxidant. Tetrahedron Lett..

[16] Valdez J., Cedillo R., Hernandez-Campos A., Yepez L., Hernandez-Luis F., Navarrete-Vazquez G., Tapia A., Cortes R., Hernandezc M., Castilloa R. (2002). Synthesis and antiparasitic activity of 1*H*-benzimidazole derivatives. Bioorg. Med. Chem. Lett..

[17] Porcari A.R., Devivar R.V., Kucera L.S., Drach J.C., Townsend L.B. (1998). Design, synthesis, and antiviral evaluations of 1-(substituted benzyl)-2-substituted-5,6-dichlorobenzimidazoles as nonnucleoside analogues of 2,5,6-trichloro-1-(beta-d-ribofuranosyl)benzimidazole. J. Med. Chem..

[18] Ravindra K.C., Vagdevi H.M., Vaidya V.P. (2008). Synthesis and antimicrobial activity of novel naphtho[2,1-*b*]furo-5*H*-[3,2-*d*][1,3,4]thiadiazolo[3,2-*a*]pyrimidin-5-ones. Arkivoc.

[19] Hubschwerlen C., Pflieger P., Specklin J.L. (1992). Pyrimido[1,6-*a*]benzimidazoles: a new class of DNA gyrase inhibitors. J. Med. Chem..

[20] Rangarajan M., Kim J.S., Jin S., Sim S.P., Liu A., Pilch D.S., Liu L.F., LaVoie E.J. (2000). 2″-Substituted 5-phenylterbenzimidazoles as topoisomerase I poisons. Bioorg. Med. Chem..

[21] Shi D.F., Bradshaw T.D., Wrigley S. (1996). Antitumor benzothiazoles. 3. Synthesis of 2-(4-aminophenyl)benzothiazoles and evaluation of their activities against breast cancer cell lines in vitro and in vivo. J. Med. Chem..

[22] Yun H., Yang J., Baogen W., Risen L., Swayze E.E. (2004). Synthesis and biological evaluations of novel benzimidazoles as potential antibacterial agents. Bioorg. Med. Chem. Lett..

[23] Zhan Z.H., Liang Y., Yong W.M. (2007). An expeditious synthesis of benzimidazole derivatives catalyzed by Lewis acids. Catal. Commun..

[24] Oren I.Y., Yalcin I., Sener E.A., Ucarturk N. (2004). Synthesis and structure-activity relationships of new antimicrobial active multisubstituted benzazole derivatives. Eur. J. Med. Chem..

[25] Gardiner J.M., Colin R., Loyns A.B., Khan A., Mahmood N. (1995). Synthesis and HIV-1 inhibition of novel benzimidazole derivatives. Bioorg. Med. Chem. Lett..

[26] El Ashry E.H., Abdel Hamide H., Ramadan E., Hagar M. (2004). Microwave Irradiation for Accelerating each Step for the Synthesis of 1,2,4-Triazino[5,6-*b*]indole-3-thiolsand their Derivatives from Isatin and 5-Chloroisatin. Synlett.

[27] Ragaini F., Ventriglia F., Hagar M., Fantauzzi S., Cenini S. (2009). Synthesis of Indoles by Intermolecular Cyclization of Unfunctionalized Nitroarenes and Alkynes: One-Step Synthesis of the Skeleton of Fluvastatin. Eur. J. Org. Chem..

[28] Aboelnaga A., Hagar M., Soliman S.M. (2016). Ultrasonic Synthesis, Molecular Structure and Mechanistic Study of 1,3-Dipolar Cycloaddition Reaction of 1-Alkynylpyridinium-3-olate and Acetylene Derivatives. Molecules.

[29] Schmidt R., Fuhrmann S., Wondraczek L., Stolle A. (2016). Influence of reaction parameters on the depolymerization of H_2_SO_4_-impregnated cellulose in planetary ball mills. Powder Technol..

[30] Rightmire N.R., Hanusa T.P. (2016). Advances in organometallic synthesis with mechanochemical methods. Dalton Trans..

[31] Margetić D., Štrukil V. (2016). Practical Considerations in Mechanochemical Organic Synthesis. Mechanochemical Organic Synthesis.

[32] Trivedi R., De S.K., Gibbs R.A. (2006). A convenient one-pot synthesis of 2-substituted benzimidazoles. J. Mol. Cat. A Chem..

[33] Thakuria H., Das G. (2008). An expeditious one-pot solvent-free synthesis of benzimidazole derivatives. Arkivoc.

[34] Shen M., Cai C. (2007). Ytterbium perfluorooctanesulfonates catalyzed synthesis of benzimidazole derivatives in fluorous solvents. J. Fluorine Chem..

[35] Babu K.S., Sankar T.R., Latha J., Babu B.R., Kumari M.S. (2013). Synthesis and Antibacterial activity of some Novel 1-piperidin-4-yl-1,3-dihydro-2*H*-benzimidazol-2-one analogs. J. Appl. Chem..

[36] Mavrova A.T., Denkova P., Tsenov Y.A., Anichina K.K., Vutchev D.I. (2007). Synthesis and antitrichinellosis activity of some bis(benzimidazol-2-yl)amines. Bioorg. Med. Chem..

[37] El Ashry E.H., El Kilany Y., Nahas N.M., Barakat A., Al-Qurashi N., Ghabbour H.A., Fun H. (2016). Synthesis and Crystal Structures of Benzimidazole-2-thione Derivatives by Alkylation Reactions. Molecules.

